# The Effect of Vitamin A Supplementation on Biochemical Parameters in Multiple Sclerosis Patients

**DOI:** 10.5812/ircmj.3480

**Published:** 2013-03-05

**Authors:** Sima Jafarirad, Fereydoon Siassi, Mohammad-Hossein Harirchian, Reza Amani, Sama Bitarafan, Aliakbar Saboor-Yaraghi

**Affiliations:** 1 Department of Nutrition and Biochemistry, School of Public Health, Tehran University of Medical Sciences, Tehran, IR Iran; 2 Iranian Center for Neurological Research, Tehran University of Medical Sciences, Tehran, IR Iran; 3 Department of Nutrition, School of Paramedicine, Jundishapour University of Medical Sciences, Ahvaz, IR Iran

**Keywords:** Vitamin A, Multiple Sclerosis, C-reactive Protein

## Abstract

**Background:**

Vitamin A has different functions in the body and after being converted to acid form; it can play many roles in immune system regulation. Therefore, this vitamin can be used as a supplement in the treatment of diseases, such as cancer and autoimmune diseases. Vitamin A is a fat-soluble compound and its long-term consumption in high doses can have some adverse effects.

**Objective:**

The current study aimed to investigate the possible complications and find solutions to minimize the adverse effects.

**Patients and Methods:**

This study was a double blind randomized clinical trial. In the main study, vitamin A (as retinyl palmitate) was given to 35 multiple sclerosis (MS) patients in order to regulate their immune system with a dose of 25000 IU/day for a period of six months. To investigate the possible biochemical complications, lipid profiles, fasting blood sugar (FBS), liver enzymes, and C-reactive protein (CRP) were tested.

**Results:**

Vitamin A did not have a significant difference in lipid profiles, FBS and liver enzymes between the two groups receiving vitamin A and the placebo, but CRP increased in patients who were taking vitamin A, 1.65±0.43 (mg/L) and 2.88±0.67, (Mean±SEM), before and after the intervention respectively (P=0.029), and statistical analysis showed significant differences with the group receiving placebo (P=0.011) and CRP level in vitamin A group was 1.3 mg/L more than those of the placebo group after intervention (P=0.011).

**Conclusions:**

Considering that no significant difference was found in the proven vitamin A side effects, due to the increase in CRP, frequent clinical and biochemical controls are required along with vitamin A supplementation.

## 1. Background

Vitamin A is an essential nutrient that is found mostly in animal products as retinyl esters and in fruits and vegetables as the form of carotenoids (the precursor of vitamin A) (1). This vitamin has various roles in a human’s body (2) such as increasing the number and effect or functions of natural killer cells, neutrophils, B and Th2 cells (3-7). One of the effects of vitamin A on T-cells is the inhibition of Th1 and the increase in Th2 differentiation (3). Multiple sclerosis (MS) is one of the most common diseases of the central nervous system (CNS) dysfunction, by which 5-15 cases per each 10000 of Caucasian are affected (8). There is strong evidence that MS is caused due to the T-cell response to myelin antigens (9).According to the effect of vitamin A on the immune system and Th1 and Th2 cells (3) there might be the possibility of using this vitamin as the supplement in the treatment of MS autoimmune disease (10). Besides the positive effect of vitamin A on immune system, this vitamin has a long biological half-life with a relatively rapid absorption and slow clearance that can lead to problems in people using it for long-term periods (11). For example, in a study using etretinate, a synthetic retinoid, caused increases in serum triglyceride, cholesterol and liver enzymes levels (12). Along the main study on the effect of vitamin A supplementation on MS patients’ immune system, biochemical and inflammation factors have also been studied in patients who participated in the current study.

## 2. Objectives

The current study aimed to investigate the possible complications and find solutions to minimize the adverse effects.

## 3. Patients and Methods

### 3.1. Participants 

The current study was a double blind randomized clinical trial and The Ethics Committee of Tehran University of Medical Sciences approved the study. Because the main study focused on vitamin A effects on immune system function, IL-4 was used to estimate the sample size and 15 subjects were determined in each group (totally 30) but 35 MS patients participated in this project (9 men and 26 women). The inclusion criteria during this study were as follows: all patients in relapsing-remitting phase, using Interferon beta-1a (Avonex) as a treatment and patients in the range of 20-45 years old. The exclusion criteria were as follows: other autoimmune diseases, pregnancy and lactation, smoking and hypersensitivity to vitamin A compounds. All patients were under the guidance of two neurologists from the teaching hospital affiliated to Tehran University of Medical Sciences and participated in the current study after consultation with their own physicians. All of them filled the consent form. 25000 IU/day vitamin A, as the form of retinyl palmitate, (Zahravi, Tabriz, Iran) or placebo was given to subjects at the beginning and after 3 months (the period of supplementation was 6 months). Subjects, who had consumed less than 90% (18 caps) of supplements at the end of 6 months intervention, were labeled as being non-compliant (but all of the patients consumed more than 90% of supplements). Vitamin A intake from food sources was assessed with 24- hour food recall questionnaire. There was no significant difference regarding age, sex and amount of vitamin A intake from food sources between the two groups at the baseline of the study. Patients referred to their neurologist to evaluate clinical side effects of vitamin A treatment.

### 3.2. Sample Blood Collection and Experiments

Fasting blood samples were collected before supplementation and were transferred to sterile (for other cell culture experiments) Heparin tubes. Then, the blood plasma was separated and stored in -70ºC. Blood samples were taken from the participants at the end of six months supplementation and stored at -70º C again. When the blood sample was collected from the last subject at the end of supplementation period, the patients’ plasma was tested to determine fasting blood sugar (FBS), lipid profiles, i.e. triglyceride (TG), cholesterol and high density lipoprotein (HDL), two liver enzymes i.e. Alanine transaminase (ALT) and aspartate transaminase (AST), and C-reactive protein (CRP) inflammation factor. Lipid profiles, FBS, ALT, and AST were determined with colorimetric method by Cobas Mira Plus (Roche, Switzerland). Low density lipoprotein (LDL) was measured with Friedewald equation (13). Immunoturbidimetric method was used to determine high sensitive-CRP (hs-CRP) (Pars Azmoon, Karaj, Iran).

### 3.4. Statistical Analysis

Independent samples t-test was used to determine the differences in biochemical and inflammation factors between the two vitamin A and placebo groups. Data in each group were analyzed with paired t-test, before and after the supplementation period. P < 0.05 was considered as significant level. SPSS software (version 17.0) was used for data analysis.

## 4. Results

FBS, lipid profiles including TG, cholesterol, LDL, HDL and their ratio were studied in the patients between the two vitamin A and placebo groups before and after supplementation period. When we compared lipid profiles, FBS and liver enzymes before or after intervention, there was no statistically significant difference between the two groups. Despite the fact that higher levels of TG had decreased in the patient group receiving vitamin A compared to those of the placebo group (20 mg/dl and 6.2 mg/dl respectively), this reduction was not significant (P = 0.321) (Table 1). Cholesterol declined in the subjects who received vitamin A (P = 0.38), but increased in the placebo group (P = 0.504). Cholesterol level in vitamin A group was 20.7 mg/dl less than the placebo group after intervention (P = 0.273). However, the difference between the two groups was not significant. LDL remained approximately intact in the group receiving vitamin A (about 0.1 mg/dl decrease), and a 0.64 mg/dl increase was observed in the placebo group though not significant (Table 1).

**Table 1.** Lipid Profilesand Fasting Blood Sugar in Subjects in Two Vitamin A and placebo groups

Profiles	Vitamin A Group, (n = 18), Mean ± SEM		Placebo Group, (n = 17), Mean ± SEM		P value
	Before	After	Before	After	
**FBS, mg/dl**	91.94±2.25	90.22±3.3	92.41±1.98	87.17±1.3	0.186
	P=0.445		P=0.001		
**TG, mg/dl**	133.44±21.11	113.33±17.77	103.17±11.34	96.88±10.85	0.321
	P=0.104		P=0.364		
**Cholesterol, mg/dl**	155.5±7.49	149.38±6.22	157.05±4.21	160.05±4.53	0.273
	P=0.38		P=0.504		
**LDL, mg/dl**	85.05±5.26	84.94±4.24	87.82±3.87	88.47±3.52	0.897
	P=0.982		P=0.841		
**HDL, mg/dl**	44.38±2.41	45±2.78	46.17±2.43	48.7±2.88	0.41
	P=0.721		P=0.123		
**LDL/HDL, ratio**	2.01±0.154	1.97±0.15	1.99±0.139	1.91±0.14	0.811
	P=0.679		P=0.346		
**Cholesterol/HDL, ratio**	3.67±0.26	3.48±0.23	3.54±0.2	3.45±0.21	0.627
	P=0.274		P=0.454		

**Figure 1.**
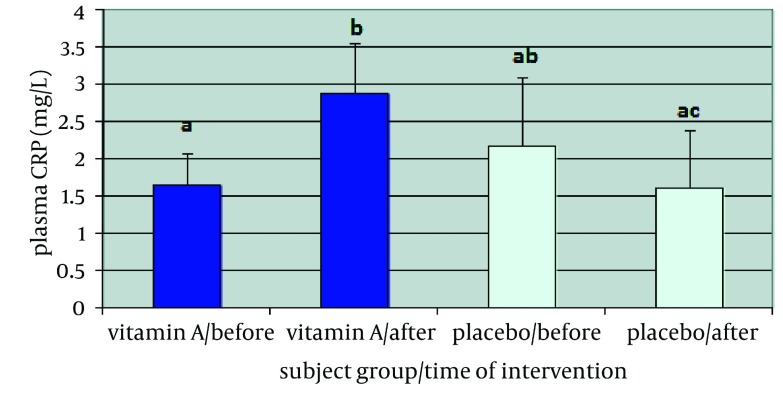
Mean ofhs-CRP in patients who received vitamin A and placebo before and after of supplementation period. Data are shown as Mean ± SEM. Different letters shows statistically significant P-value

**Figure 2.**
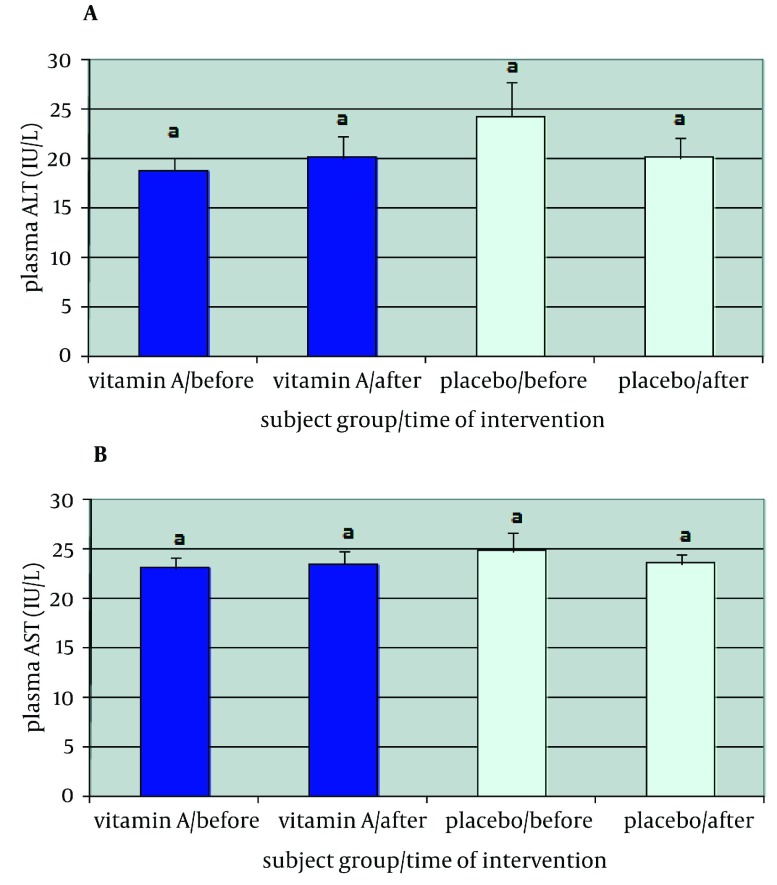
(a) Mean of ALT in plasma in two groups of patients who participated in this study (b) Mean of AST in plasma in two groups of patients Data are shown as Mean ± SEM. The same letters shows no statistically significant P value

High sensitive-CRP (hs-CRP) status of patients participating in this study is shown in Figure 1. hs-CRP levels had a rise of nearly 1.2 mg/L in patients using vitamin A as a supplement (P = 0.029) and decreased about 0.5 mg/L in subjects using placebo (P = 0.186). Significant statistical difference was observed between the two groups, hs-CRP plasma level in vitamin A group was 1.3 mg/L more than the placebo group post intervention (P = 0.011). Levels of this inflammation factor showed a significant difference in vitamin A group before and after supplementation (P = 0.029), but this difference was not significant in placebo group (P = 0.186). ALT liver enzyme enhanced approximately to 1.3 IU/L in vitamin A group (P = 0.433) whereas in the placebo group had a 4.2 IU/L decrease (P = 0.092). No significant difference was shown between the two groups before and after supplementation. ALT level in vitamin A group was 5.5 IU/L more than that of the placebo group after supplementation (P = 0.061). This difference in each group was not also significant before and after six-month supplementation period (Figure 2a). AST was increased 0.4 IU/L in vitamin A group (P = 0.778) and decreased 1.3 IU/L in placebo receiving subjects (P = 0.486). Independent samples t-test did not show significant difference between the two groups before and after the supplementation period (P = 0.748). Data analysis with paired sample t-test did not show significant difference in each group before and after the six-month period (P = 0.778 and P = 0.486 in vitamin A and placebo group respectively) AST level in placebo group was 1.7 IU/L less than that of the vitamin A group after intervention (P = 0.46) (Figure 2b).

## 5. Discussion

Vitamin A is a fat-soluble compound whose essential role in vision, reproduction and morphogenesis, growth and differences has been investigated (14). This vitamin is also involved in the regulation of gene expression, for which retinol must be converted to its carboxylic acid form: all-trans retinoic acid (ATRA) or 9-cis retinoic acid (9-cis RA) which are ligands for two families of retinoid receptor i.e. retinoic acid receptor (RAR) and retinoid X receptor (RXR) (15). More than 532 genes have been identified as retinoic acid target genes (16). Metabolites of vitamin A can modulate particular functional aspects of immune responses, such as Th1/Th2 balance, regulatory T-cells (Treg) and Th17 differentiation (17). As a result, using this vitamin could have a regulatory role in autoimmune diseases, such as MS (10). However, taking too much of vitamin A for a long time might be risky (11). Since this vitamin is stored in the liver (14)and receiving high levels of vitamin A stimulates gluconeogenesis and protein turnover, it could induce hepatotoxicity in low protein diets (18). However, most of the patients participating in this study were from high social class and their protein intake was enough (data not shown). AST and ALT enzymes were used to assess liver function. These two enzymes had increased in the group receiving vitamin A, while both enzymes were in the normal range (0-41 IU/L) in the two groups. In this survey, cholesterol level in patients receiving vitamin A decreased, but there was an increase in placebo recipients with no significant difference. Despite this insignificant difference, cholesterol and TG reduction in these patients have been positive unexpected results (19) since in other studies on isotretinoin, etretinate, and other metabolites of vitamin A, higher levels of cholesterol and TG were observed in pharmacologic dosage of vitamin A supplement (20-24). This increase in plasma TG levels by retinoid is considered as a result of escalation in apo C-III level (25). In a study, carried out on CaCo-2 cells, retinoic acid (RA) had no effect on the synthesis of apo A-I and the HDL, LDL and other apos (A-IV, B-48, B-100 and E) production (26). If the levels of Apo lipoproteins were measured in this study, there could be a more precise judgment on the status of lipid profile. The significant point of this study was the increase in CRP level among vitamin A supplement consumers compared with the consumers in the placebo group. It seems that the decreasing effect of vitamin A on CRP is through its precursors i.e. carotenoids (27, 28) not it’s preformed. In another study on men who were healthy nonsmokers, with consumption of 8 servings of fruit and vegetables rich in carotenoids per day, hs-CRP level reduced compared with those taking two servings per day (29). As the inflammatory phase proteins can be induced by certain cytokines, such as IL-1, IL-6, and TNF-α (30) and the fact that retinoid decreases the secretion of IL-1β and TNF-α, (31, 32) the increase of hs-CRP was an unexpected result. However, hs-CRP level in these patients had not exceeded the normal range. Notably in a survey on pre-school children in Ghana, high doses of vitamin A- every 4 months for one year- elevated CRP in children with gastrointestinal infections with vomiting (33). In contrast, after 5 months, no significant effect in CRP level was observed in 3-to-6-year old Indonesian children taking an oral high dose of vitamin A (200000 IU)(34). Despite the investigation of vitamin A side effects in various researches, the dose range of this vitamin that can create problems for different people is between 40000-50000 IU/day; (11) vitamin A dosage in this study was 25000 IU/day. Given the rise in CRP in these patients (despite being in the normal range) and the positive effects of this vitamin on immune system, vitamin A supplementation has to be conducted with caution and frequent clinical checkups in MS patients.
